# Supplementary material for: Impact of Maternal Mediterranean-Type Diet Adherence on Microbiota Composition and Epigenetic Programming of Offspring
Source: Nutrients. 2023 Dec 22;16(1):47. doi: 10.3390/nu16010047 (PMC10780434; doi:10.3390/nu16010047)
Supplement: Supplementary file 1 [file nutrients-16-00047-s001.zip › nutrients-2755297-supplementary.pdf]

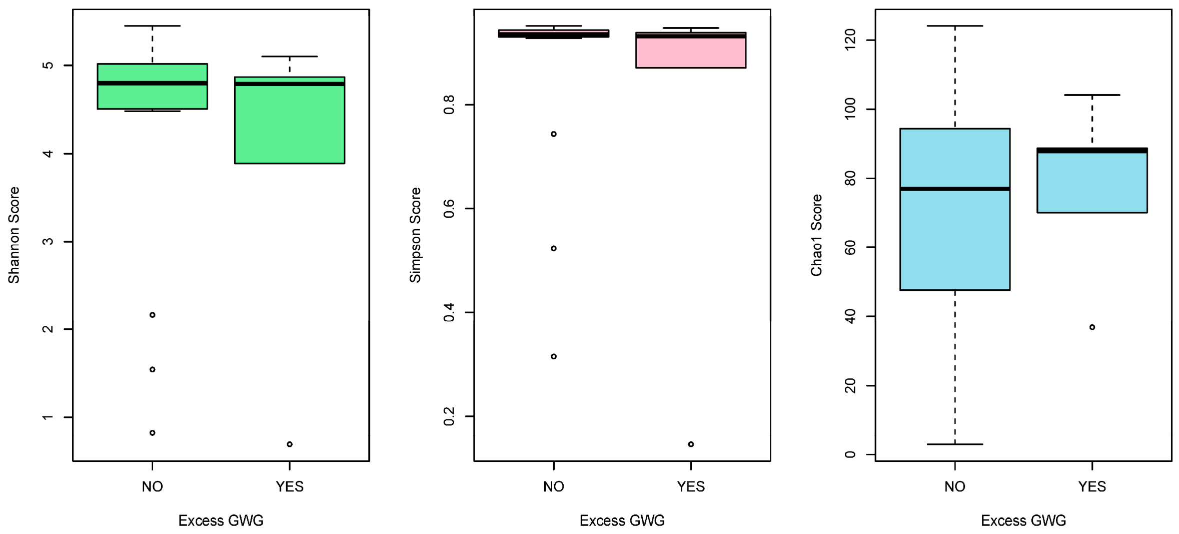

Figure S1. Alpha diversity (Chao 1, Shannon, and Simpson) comparison between participants with excess gestational weight gain. There were no statistically significant differences between the two groups (*p* values: Chao1 = 0.68, Shannon = 0.71, Simpson = 0.60).

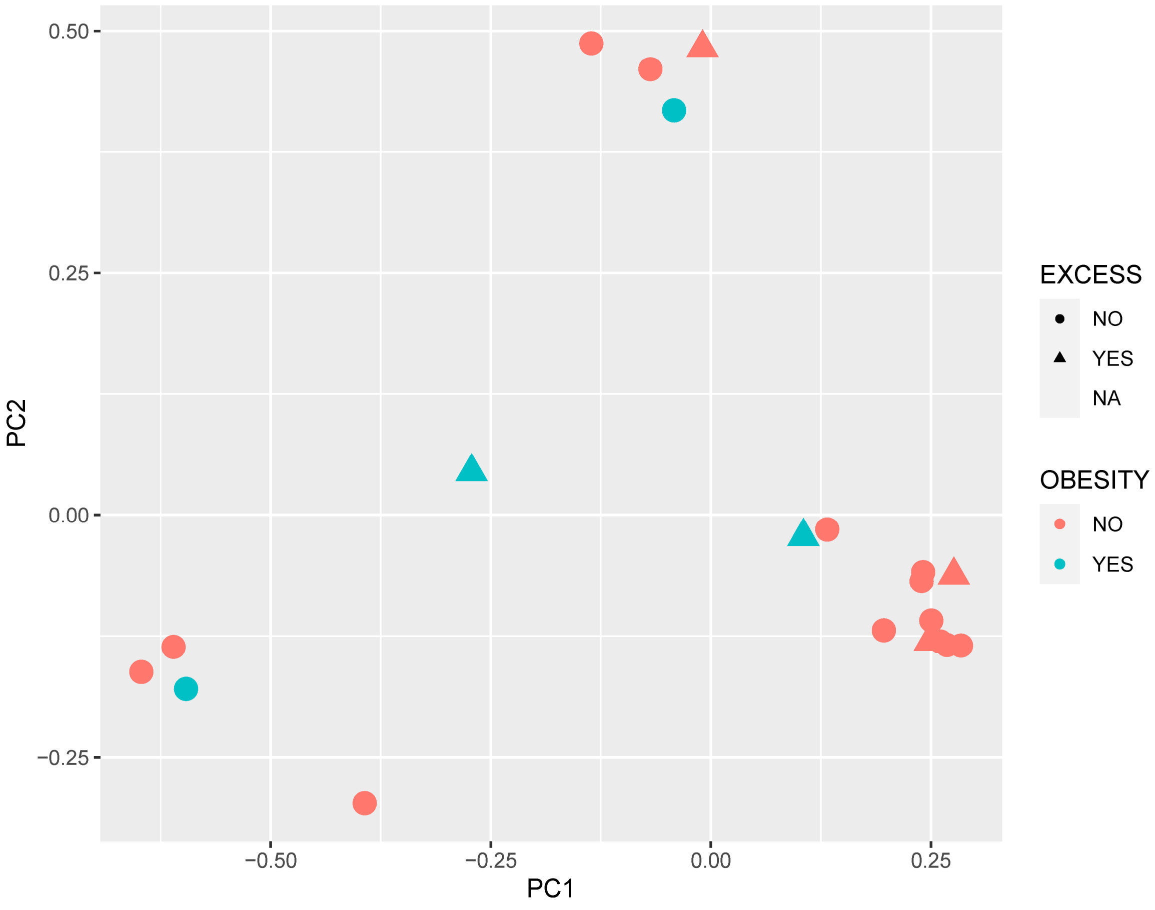

Figure S2. Beta diversity plot to evaluate Bray-Curtis distance among Obese vs. non-obese participants and those who had excess gestational weight gain and those who did not. No similar grouping was noted for either covariate.
